# Identification lodging degree of wheat using point cloud data and convolutional neural network

**DOI:** 10.3389/fpls.2022.968479

**Published:** 2022-09-27

**Authors:** Yunlong Li, Baohua Yang, Shuaijun Zhou, Qiang Cui

**Affiliations:** School of Information and Computer, Anhui Agricultural University, Hefei, China

**Keywords:** UAV image, point cloud, classification, wheat, lodging

## Abstract

Wheat is one of the important food crops, and it is often subjected to different stresses during its growth. Lodging is a common disaster in filling and maturity for wheat, which not only affects the quality of wheat grains, but also causes severe yield reduction. Assessing the degree of wheat lodging is of great significance for yield estimation, wheat harvesting and agricultural insurance claims. In particular, point cloud data extracted from unmanned aerial vehicle (UAV) images have provided technical support for accurately assessing the degree of wheat lodging. However, it is difficult to process point cloud data due to the cluttered distribution, which limits the wide application of point cloud data. Therefore, a classification method of wheat lodging degree based on dimensionality reduction images from point cloud data was proposed. Firstly, 2D images were obtained from the 3D point cloud data of the UAV images of wheat field, which were generated by dimensionality reduction based on Hotelling transform and point cloud interpolation method. Then three convolutional neural network (CNN) models were used to realize the classification of different lodging degrees of wheat, including AlexNet, VGG16, and MobileNetV2. Finally, the self-built wheat lodging dataset was used to evaluate the classification model, aiming to improve the universality and scalability of the lodging discrimination method. The results showed that based on MobileNetV2, the dimensionality reduction image from point cloud obtained by the method proposed in this paper has achieved good results in identifying the lodging degree of wheat. The F1-Score of the classification model was 96.7% for filling, and 94.6% for maturity. In conclusion, the point cloud dimensionality reduction method proposed in this study could meet the accurate identification of wheat lodging degree at the field scale.

## Introduction

Wheat is one of the three major food crops in the world, and its output directly affects food security. Among them, wheat growth is a key factor affecting wheat yield (Hu et al., 2020). The height of wheat is one of the common phenotypic parameters used to assess its growing status. On the one hand, the height changes of wheat in different periods, especially the height changes caused by wheat lodging in the middle and late stages, provide reference data for wheat health assessment and yield estimation (Piñera-Chavez et al., 2020). On the other hand, monitoring and diagnosis of wheat lodging degree is an important basis for field risk assessment and precise management. Therefore, large-scale and accurate judgment of the degree of wheat lodging is of great significance for field management, yield estimation, and damage assessment.

In recent years, the development of remote sensing technology provides technical support for the rapid and accurate acquisition of image information and spatial information of large-scale farmland. unmanned aerial vehicle (UAV) remote sensing technology provides a new solution for the acquisition and analysis of high-throughput phenotypic information of field crops due to its powerful flexibility, efficiency, and simplicity (Gracia-Romero et al., 2019; Zeybek and Şanlıoǧlu, 2019). In particular, the height of field crops, which can be acquired by carrying different imaging sensors, as an indicator of phenotypic traits, is one of the key steps to improve the accuracy and efficiency of crop growth monitoring (Malachy et al., 2022). Among them, the efficient, non-destructive, and high-precision UAV-LiDAR can achieve real-time and comprehensive data collection (Zhao et al., 2021). Studies have shown that UAV-LiDAR was used to obtain 3D point cloud information of ground objects, which generates a digital elevation model to obtain plant height of crops. This method has been applied to various crops, such as vegetable wheat (Guo et al., 2019), corn (Zheng et al., 2015), rice (Tilly et al., 2014; Phan and Takahashi, 2021), soybean (Luo et al., 2021), etc. The above results could better realize the analysis of crop phenotype indicators. However, complicated processing procedures and expensive instruments limited the development of UAV-LiDAR remote sensing monitoring. Therefore, it is of great research value for lodging identification and crop growth assessment how to use remote sensing technology to quickly and accurately obtain crop growth information.

With the development of sensors, digital cameras have promoted the convenience and practicability of UAV high-throughput phenotyping platforms due to their low cost, lightness, and high resolution. For crop height monitoring, two common types of data are extracted from overlapping aerial images acquired by consumer digital camera, including digital orthophoto map (DOM) and digital surface model (DSM). Such as the height of wheat (Villareal et al., 2020), rice (Liu H. et al., 2018), maize, cotton and sorghum (Wu et al., 2017), and potatoes (Li et al., 2020). In addition, crop surface models (CSMs) are models formed by digitizing the morphology of plants. Therefore, it contains information about the overall shape of the plant and is often used to estimate the height of the plant. For example, Bendig et al. (2015) used CSMs to estimate the height of barley, and Volpato et al. (2021) used CSMs to extract the height information of wheat. The height and growth information of crops can also be obtained by using the digital elevation model (DEM) of the terrain of the experimental area. For example, the DEM model has been successfully used to estimate the height of cotton (Feng et al., 2019), and sugarcane (Sumesh et al., 2021). Currently, there is no generally accepted consensus on which method of DSM, CSM, or DEM model works better. Therefore, the extraction of crop height information still faces many challenges.

In fact, the point cloud data can obtain the information of the horizontal and vertical dimensions of the lodging crops at the same time, which can effectively reflect the height changes of the crops, especially the lodging degree of the crops. Although those Hu et al. (2021) used deep learning to process point cloud data successfully achieved quantitative analysis of lodging degree. However, disorder and irregularity make 3D point cloud data difficult to process (Guo Y. et al., 2021). Many scholars have proposed some deep learning methods to directly process point clouds. For example, PointNet point cloud learning network (Qi et al., 2017a), PointNet++ (Qi et al., 2017b), MV3D (Chen et al., 2017), 3D-BoNet (Yang et al., 2021a). However, the models mentioned above still have some issues such as low accuracy and poor robustness. Therefore, it is necessary to study methods about point cloud data processing.

Due to the sparse and unstructured characteristics of point cloud data, indirect processing of 3D scattered data will reduce the difficulty and complexity of point cloud processing (Liu and Bai, 2018). Studies have shown there have been many attempts to transform point cloud data into other forms. For example, Su et al. (2015) proposed to map point clouds to 2D images, and convolutional neural network (CNNs) were used to classify images. Zhou and Tuzel (2018) proposed to rasterize point clouds into voxels, and the 3D CNN was used to extract the local features of the voxel grid. Although point cloud homogenization has been achieved in the above research. However, to homogenize the point cloud data, it is necessary not only to reduce the dimensionality of the point cloud data, but also to apply spatial interpolation and spatial fitting methods to predict the data values of some blank locations. Of course, the Hotelling transform has the potential to solve the above issues (Chen and Chung, 2010). Therefore, the Hotelling transform method was used to reduce the dimensionality of the 3D point cloud data into a 2D image in this study, which provided a new idea for point cloud data processing.

CNN, as one of the commonly used deep learning methods, has excellent performance in a variety of image processing tasks, due to its local connection, and weight sharing (Yang et al., 2021b). Therefore, a classification method of wheat lodging degree based on CNN was proposed using images obtained by dimensionality reduction from point cloud data in this study. The self-built wheat lodging dataset was used to evaluate the performance of the method, aiming to improve the robustness of the lodging classification method. The purpose of this research is to (1) propose a point cloud dimensionality reduction method, which realized the conversion of 3D point cloud data into 2D image based on Hotelling transform and interpolation method, aiming to reduce the complexity of point cloud data processing, (2) propose a method for wheat lodging identification with point cloud data extracted from UAV images, aiming to improve the accuracy of identification, and (3) identify different lodging degrees of wheat using different CNN models, aiming to verify the robustness of the proposed method.

## Materials and methods

### Data collection

#### Acquisition wheat lodging angle and lodging area

Data was collected on May 7 and May 17, 2021 in the National Modern Agriculture Demonstration Zone (31°29′26″N, 117°13′46″E) in Guohe Town, Lujiang County, Anhui Province. On April 30, 2021, Lujiang County experienced hail, heavy rainfall, and strong winds of magnitude 4–5, resulting in varying degrees of wheat lodging in the study area. Field surveys and UAV monitoring were carried out on the wheat fields (filling stage, maturity stage) in the study area. Filling and maturity are the key periods to determine the grain weight of wheat, which directly affect the yield of wheat at harvest.

The wheat field was defined as a 3.8 m × 7.8 m plot, and the lodging area and lodging angle were measured for each wheat plot. The length and width of each wheat plot were measured and used to calculate the total lodging area of all the plots. To calculate the lodging angle, the sloping and vertical heights of the lodging wheat in the plot were measured using a tape measure, and 3–5 samples were measured in the same observation plot, and the average value of the lodging angles of the multiple samples were calculated as the lodging angle of the observation plot. A total of 360 wheat plots were measured.

#### Determination of wheat lodging degree

To evaluate the lodging degree, the lodging index (LI) was used to evaluate the lodging degree of wheat. The value of the lodging index is between [0, 1]. Among them, “0” represents normal growing wheat, “1” represents complete lodging, and the calculation formula is shown in the formula (1)-(2).


(1) LI=LA×LR

(2) $$\mathrm{LA}=\frac{2\,\theta }{\pi }=\frac{\pi -\arcsin\frac{h_{2}}{h_{1}}\times \frac{\pi }{90}}{\pi }$$

**FIGURE 1:**
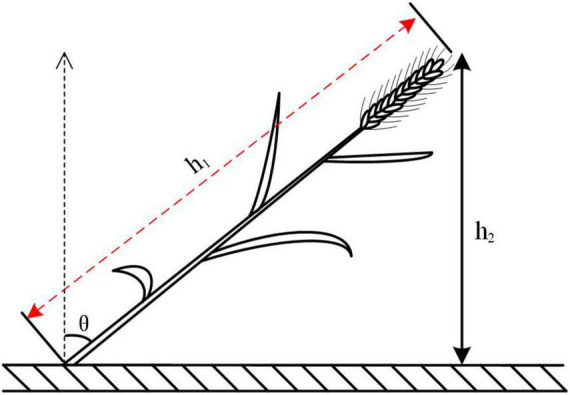
Diagram of angle of lodging.

Among them, h_1_ represents the actual height of wheat in the experimental plot and h_2_ represents the canopy height of the experimental plot. The larger the value of the lodging angle, the more serious the lodging of the wheat. θ represents the lodging angle, as shown in Figure 1.

Lodging ratio (LR) (0–100%) is the proportion of the lodging area in the total area of the wheat plot. LR can reflect the change of the lodging area in the horizontal direction. The larger the value of lodging rate, the more serious the lodging of wheat. The lodging degree of the wheat field was determined according to the lodging rate of the plot based on the double-threshold strategy of normal statistical theory. The specific steps are as follows: calculate the mean (μ) and standard deviation (α) of the lodging index of the wheat plot, and then divide the lodging index into four parts, namely [0, μ–α], [μ–α, μ], [μ, μ+α], and [μ+α, 1], corresponding to non-lodging, slight lodging, moderate lodging, and severe lodging, respectively. The mean value (μ) of lodging index of 360 wheat plots was calculated to be 0.40 and the standard deviation (α) was 0.274. Therefore, the lodging indices of different lodging degrees were determined as: non-lodging [0, 0.126], slight lodging [0.11, 0.40], moderate lodging [0.40, 0.674], severe lodging [0.674, 1]. Thus, the ground truth of different lodging degrees of wheat in different growth periods were obtained.

#### Acquisition and normalization of point cloud data

To obtain the original point cloud of the wheat field, Agisoft PhotoScan software was used to process the RGB image of the UAV. Specifically, the generation of the original point cloud in the study area is based on the structure from motion algorithm, which is used to process the input UAV images with the corresponding position and orientation system (POS), and feature point information. The purpose is to restore the spatial location information of the corresponding image feature points. However, affected by the undulation of terrain, the original point cloud of wheat field obtained after processing by Agisoft PhotoScan software still has the problem of elevation deviation. Therefore, it is necessary to normalize the acquired original point cloud of the research area to obtain normalized point cloud data, which provides a data basis for dimensionality reduction of wheat point cloud, and aims to improve the accuracy of wheat lodging degree judgment.

**FIGURE 2:**
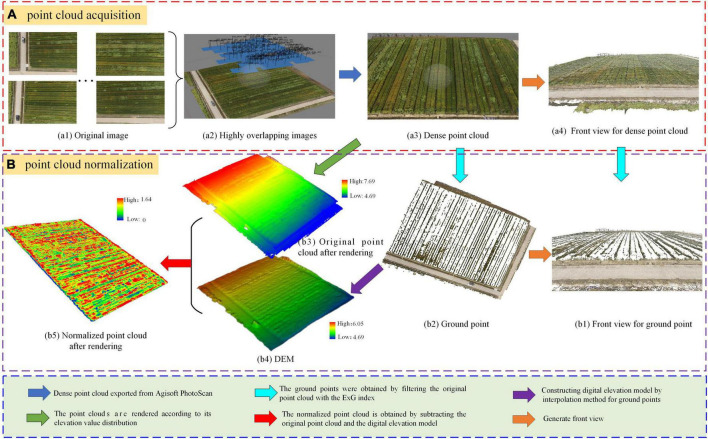
The acquisition process of normalized point cloud. **(A)** point cloud acquisition, **(B)** point cloud normalization.

The specific steps include point cloud acquisition and point cloud normalization, as shown in Figure 2. Firstly, the stitched images of wheat fields are obtained from 98 UAV images, and the density point clouds are extracted from the stitched images, as shown in Figures 2a1–a3. Secondly, the Excess Green Index (ExG) was used to obtain the ground point cloud from the density point cloud, digital elevation model (DEM) was generated by interpolation fitting. Finally, the normalized point cloud is obtained by subtracting the original point cloud and DEM, as shown in Figures 2b2–b5. And the front views for point cloud and point cloud normalization were provided, as shown in Figures 2a4,b1.

In addition, to obtain the normalized point cloud of each wheat plot, the normalized point cloud was used to realize field cutting in MeshLab software, and the experimental wheat field size 3.8 m × 78 m was cut into 180-point clouds of 3.8 m × 7.8 m, which were numbered separately and exported in TXT format to provide data for the Hotelling transform of the point cloud.

## Materials and methods

### Acquisition of dimensionality reduction image from point cloud

The initial point cloud dataset is a set of 3D data, represented by x, y, z coordinates. Therefore, it is more troublesome to process such data. On the one hand, there are many dimensions and the complexity of the data is large. On the other hand, the point cloud is scattered in space, and there is no obvious three-dimensional topology. To this end, the idea of point cloud homogenization is used to reduce the dimension of the three-dimensional point cloud, and the point cloud data after dimension reduction becomes a two-dimensional form.

Firstly, Hotelling transformation was used to realize the transformation of the point cloud coordinate system (dimension unchanged), so as to find a set of optimal orthogonal vector bases to represent the original sample data. Then, the inverse distance weighted interpolation method is used to assign the grid eigenvalues, and finally, the numerical values of the grid eigenvalues are color-rendered, so as to realize the cloud dimension reduction map of the wheat sample sites.

### Hotelling transform

The Hotelling transform is a transformation based on statistical properties, which transforms the original data set into the principal component space by finding subsets of the principal components of the data set of arbitrary statistical distribution, minimizing the cross-correlation of a single data sample. The process steps are as follows:

(1) Suppose a set of point cloud data *P*_*n*_ is represented as an 3×n dimensional matrix, each column represents a point (*x_k_*, *y_k_*, *z_k_*) in the space, *k* = 1, 2, 3…*n*, *n* represents the number of point cloud.


(3) $$P_{n}=\left[\begin{array}{cccc} x_{1} & x_{2} & \ldots & x_{n}\\ y_{1} & y_{2} & \ldots & y_{n}\\ z_{1} & z_{2} & \ldots & z_{n} \end{array}\right]$$

(4) $$m=\frac{1}{n}\,\sum_{k=1}^{n}P_{k}$$

(5) $$C=\frac{1}{n}\,\sum_{k=1}^{n}P_{k}\,P_{\text{k}}^{T}-m\,m^{T}$$

Where, *m* represents the center of gravity of the data *P*_*n*_, and *C* represents the covariance of the data *P*_*n*_.

(2) The eigenvalue decomposition is performed on the obtained covariance *C*, and the eigenvector matrix *V* and the eigenvalue matrix *D* are obtained:


(6) C×V=D×V

(3) Finally, the eigenvector *V* is in descending order according to the corresponding eigenvalues, and a local coordinate system is established with the local neighborhood gravity center *m* as the coordinate origin and the three components of the eigenvector *V* as the three coordinate axes. The point cloud data *P*_n_ is converted into a new coordinate system, and its coordinate *P*_n_ in the new coordinate system is obtained by formula (8) calculation:


(7) $$P_{n}^{{}^{\prime }}=V\times \left(P_{n}-m\right)$$

*V* represents the feature vector, $P_{n}^{{}^{\prime }}$ represents the new coordinate system.

### Grid division

To solve the problem that the point cloud is scattered and distributed, which makes it difficult to describe the characteristics, the regularization of the point cloud is realized based on the division of the grid. The size of the grid is determined according to the length range and width range of the points of the wheat field. The grid is divided according to formula (8):


(8) $$r\,o\,w\,s=\frac{\Delta \,x}{d},c\,o\,l\,s=\frac{\Delta \,y}{d}$$

where *cols* and *rows* represent the number of grid lengths and grid widths, respectively, Δ*y* and Δ*x* are the length range and width range of the point cloud data, respectively, and *d* is the grid spacing.

In this study, the length and width of the wheat site cloud were 7.8 and 3.8 m, respectively, and the grid spacing was set to 0.01 m. Therefore, a regular rectangular pixel grid of 780 × 380 was used to interpolate the wheat site cloud.

### Spatial interpolation of point cloud

Inverse distance weighting (IDW) is one of the most commonly used spatial interpolation methods. It is an interpolation method with the distance between the point to be interpolated and the actual observed sample point as the weight. The sample points that are closer to the point to be interpolated are given more weight, and their weight contribution is inversely proportional to the distance. The calculation formula is:


(9) $$Z=\sum_{i=1}^{m}K_{i}\,Z_{i}$$

(10) $$K_{i}=\frac{d_{i}^{-2}}{\sum_{i=1}^{m}d_{i}^{-2}}$$

(11) $$d_{i}=\sqrt{{\left(x-x_{i}\right)}^{2}+{\left(y-y_{i}\right)}^{2}}$$

In the formula, *Z* represents the estimated value of the point to be interpolated, *Z*_i_ is the measured value of the *i*-th sample point; *m* is the number of measured sample points; *K*_i_ is the contribution weight of the *i*-th sample point to the estimated value, and *d*_i_ is the distance between the *i*-th sample point (*x*_i_, *y*_i_) and the point (*x*, *y*) to be interpolated.

Therefore, color rendering is performed based on the attribute values of the grid points. In this way, the dimensionality reduction of point cloud after Hotelling transform is realized. To effectively evaluate the effect of interpolation. In this study, the following indicators were used for evaluation, including the mean absolute error (MAE), the standard deviation (SD), and the median (Median).


(12) $$\text{MAE}=\frac{1}{n}\,\sum_{i=1}^{n}\left|w_{i}\right|$$

(13) $$\text{SD}=\sqrt{\frac{1}{n}\,\sum_{i=1}^{n}w_{i}^{2}}$$

(14) $$\mathrm{Median}=\mathrm{Median}\,\left(w_{1},w_{2},w_{3}\,\begin{array}{ccc} . & . & . \end{array}\,w_{n}\right)$$

Here, ^w_i_^ is the error of the normal offset of point (*x*_i_, *y*_i_, *z*_i_).

### Classification for lodging degree

#### Technology roadmap

**FIGURE 3:**
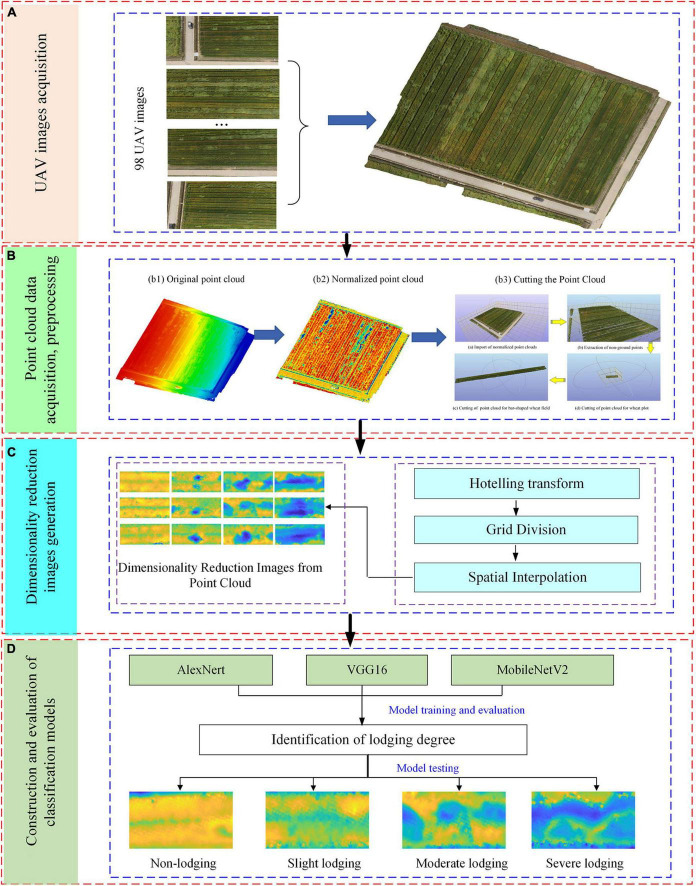
Flow chart of technical route. **(A)** UAV images acquisition, **(B)** point cloud data acquisition, preprocessing, **(C)** dimensionality reduction images generation, **(D)** construction and evaluation of classification models.

To reduce the difficulty and complexity of point cloud processing, and at the same time retain the information in the horizontal and vertical dimensions of the point cloud, a classification method of wheat lodging degree based on the dimensionality reduction image from point cloud was proposed. The specific process is shown in Figure 3.

It can be seen from Figure 3 that the specific steps include the following. Firstly, the UAV images are acquired. Secondly, point cloud data acquisition and preprocessing, including point cloud data normalization and cutting, etc. Then, the point cloud dimension reduction map is generated, including Hotelling transform, grid division, and spatial interpolation of point cloud. Finally, the classification model is constructed, and the CNN is used to classify the lodging degree of wheat, including AlexNet, VGG16, MobileNetV2, which are trained and validated with training set, and tested using the test set.

#### Classification model

In this study, AlexNet, VGG16, MobileNetV2 were used as classification models to identify the lodging degree of wheat. AlexNet is the winning network of the ISLVRC 2012 (ImageNet large scale visual recognition) competition (Krizhevsky et al., 2012). In this experiment, the target categories for predicting the lodging degree of wheat are 4 categories. Visual geometry group network (VGG) is a deep CNN proposed in 2014, which mainly uses small convolutional filters to build the network structure (Simonyan and Zisserman, 2014). The VGG16 network structure contains 16 layers, namely 13 convolutional layers, 5 pooling layers, and 3 fully connected layers. The MobileNetV2 network is an improvement based on the MobileNetV1 network (Howard et al., 2017). It follows the depthwise separable convolution (DSC) in the MobileNetV1 network, and introduces an inverted residual module containing a linear bottleneck, which can effectively improve the accuracy of image classification and detection. In addition, all models are trained and validated using a dataset consisting of bird’s-eye views which is from the point cloud data transformation. The basic parameters of the three models are compared in Table 1.

**TABLE 1:** Comparison of model parameters.

Modle name	Input_size	Number of parameters
AlexNet	224 × 224 × 3	16630440
VGG 16	224 × 224 × 3	138357544
MobileNetV2	224 × 224 × 3	3504872

#### Classification functions

To adapt to the four classification tasks (non-lodging, slight lodging, moderate lodging, and severe lodging) of wheat lodging in this study, the classifier Softmax of the above three CNN models is changed to four targets. The Softmax classifier is suitable for the processing of multi-classification target tasks, and converts each type of output into a value between [0, 1], making the sum of all classification probabilities to be 1.

Taking the output of the ^i^-th node of the neural network as an example, the mathematical formula definition of the Softmax function is given:


(15) $$\mathrm{Soft}\,\max\,\left(z_{i}\right)=\frac{e^{z^{i}}}{\sum_{c=1}^{C}e^{z}\,C}$$

where ^z_i_^ — the output value of the *i*-th node, *c*, count variable, C, the number of output nodes, that is, the number of categories of classification.

After the above function transformation, the output value of the multi-classification can be converted into a probability distribution ranging from 0 to 1.

#### Evaluation index

To effectively evaluate the classification effect of wheat lodging degree, Accuracy and *F1-score* are used as evaluation indicators.


(16) $$\mathrm{Accuracy}=\frac{\mathrm{TP}+\mathrm{TN}}{\mathrm{TP}+\mathrm{TN}+\mathrm{FP}+\mathrm{FN}}$$

(17) $$\mathrm{Precision}=\frac{\mathrm{TP}}{\mathrm{TP}+\mathrm{FP}}$$

(18) $$\mathrm{Recall}=\frac{\mathrm{TP}}{\mathrm{TP}+\mathrm{FN}}$$

(19) $$\text{F1-score}=\frac{2\,\text{×}\,\mathrm{Precision}\,\text{×}\,\mathrm{Recall}}{\mathrm{Precision}+\mathrm{Recall}}$$

Among them, TP represents the number of positive samples predicted to be Ture, FN represents the number of samples that are predicted to be False; TN represents the number of negative samples that are predicted to be True, FP represents the number of samples that were predicted to be False.

## Results and analysis

### Generation of normalized point cloud

**FIGURE 4:**
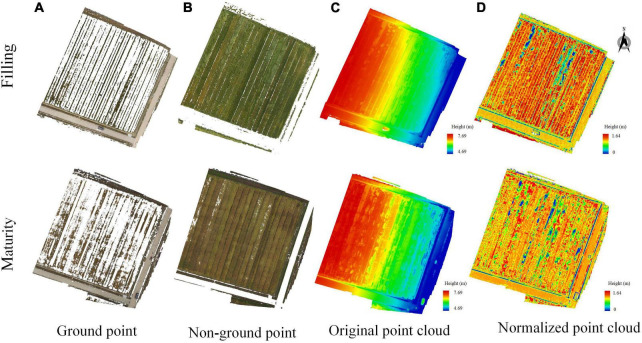
Normalized point cloud of wheat field in different periods. **(A)** ground point, **(B)** non-ground point, **(C)** original point cloud, and **(D)** normalized point cloud.

Figure 4 showed point cloud extraction and normalization of the wheat field of and maturity stages of wheat. Among them, through many experiments, ExG was set to 0.0729 to separate the ground and non-ground in the image, and the results were shown in Figures 4A,B. Figure 4C was the point cloud of the research area before normalization, and Figure 4D was the point cloud of the research area after normalization.

It could be seen from Figure 4 that the point clouds of different periods were different. For the original point cloud in Figure 4C, the west side of the wheat field grew better, and the lodging area of the wheat field was less. The eastern part of the wheat field was affected by the heavy rain, resulting in a large area of lodging of the wheat field. For the normalized point cloud in Figure 4D, which could better show the growth state of wheat. The height of the wheat field in the maturity stage was obviously lower than that in the grain filling stage, and most of them were below 0.9 m. Moreover, the area of lodging has increased significantly, and the degree of lodging has also become heavier.

### Analysis of dimensionality reduction results in different periods

**FIGURE 5:**
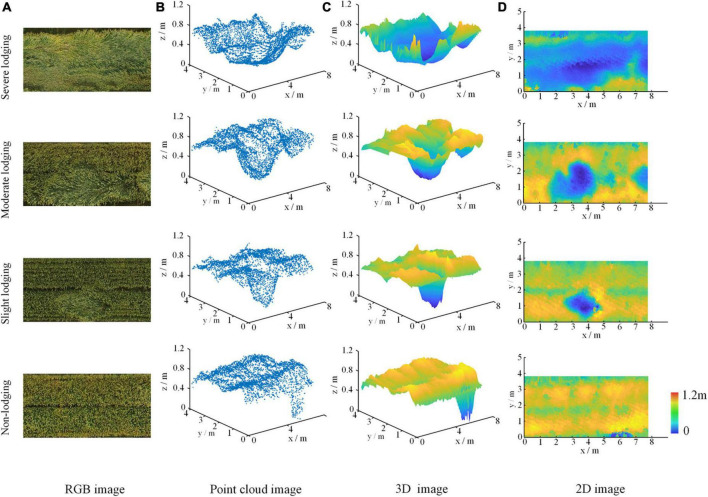
The result of point cloud dimensionality reduction of wheat field at filling stage. **(A)** RGB image, **(B)** point cloud image, **(C)** 3D image, and **(D)** 2D image.

**FIGURE 6:**
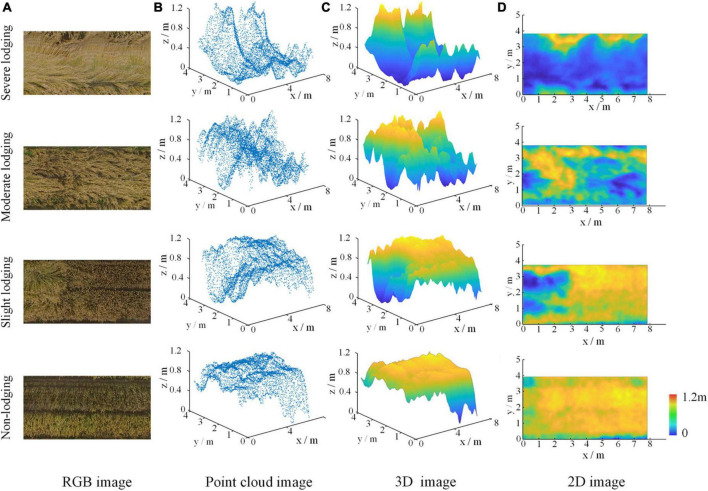
The result of point cloud dimensionality reduction of wheat field at maturity stage. **(A)** RGB image, **(B)** point cloud image, **(C)** 3D image, and **(D)** 2D image.

Figures 5, 6 showed dimensionality reduction images from point cloud of wheat fields at grain filling and maturity stages, respectively, which were obtained by an inverse distance weighted interpolation method. For the filling stage, Figures 5A,B were the RGB images and point cloud images of the wheat plots with the four different lodging degrees, respectively, and Figures 5C,D are the plots of 3D image, 2D image using distance-weighted interpolation. For the maturity stage, Figures 6A–D represented the RGB image, point cloud image, 3D images of point cloud, and 2D image of the wheat plot with the four lodging degrees, respectively.

From Figures 5B, 6B, it could be seen intuitively that no matter which period it is, the point cloud data of wheat fields had a large amount of data and are irregularly arranged. It could be seen from Figures 5C, 6C that the three-dimensional angle image of wheat field obtained by inverse distance weighted interpolation could better reflect the canopy height distribution of wheat plots with different lodging degrees. The height distribution of wheat plots with different lodging degrees was also different. Among them, the z-axis value of the non-lodging wheat plot is above 0.9 m; 0.7–0.9 m for the slightly lodging wheat plot, 0.5–0.7 m for the moderately lodging wheat plot, and below 0.5 m for the severely lodged wheat plots.

It could be seen from Figures 5D, 6D that the two-dimensional images of wheat plots with different lodging degrees after inverse distance weighted interpolation were properly smoothed, and the grid point data was relatively complete, which could reflect the distribution of wheat lodging. In particular, it was easy to compare the location and height distribution of different lodging degrees in the wheat plots from the dimensionality-reduced images. Therefore, the images obtained by point cloud dimensionality reduction could better reflect the differences in the lodging degree of wheat, and provide a data basis for judging the lodging degree of wheat.

### Classification results of lodging degree based on point cloud

#### Construction of dataset from point cloud

**FIGURE 7:**
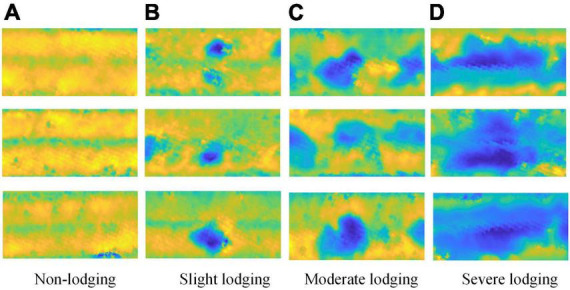
The dataset for classification of wheat lodging. **(A)** non-lodging, **(B)** slight lodging, **(C)** moderate lodging, and **(D)** severe lodging.

A total of 180 wheat plots were monitored in this study, and 360 original dimensionality-reduced images were obtained for the two periods of wheat. To improve the generalization ability of the network, random multi-angle rotations, such as 90°, 270°, horizontal flip, mirror flip, etc., which are used to augment the data of the dimensionality-reduced image of wheat from the point cloud, and 640 images are obtained. There are a total of 1,000 dimensionality reduction images from point cloud for wheat as the dataset for this experiment, which is divided into training set, validation set, and test set according to 16:4:5. Therefore, the training set is 640, the validation set is 160, and the test set is 200. In addition, there are 50 images of each lodging degree for test set. The dataset is shown in Figure 7.

#### Hyperparameter settings

The software environment for image processing and analysis experiments is based on the Windows 10 operating system, the PyTorch deep learning framework, using Python as the programming language, and using PyCharm to build models. The test hardware environment is 16 GB memory, NVIDIA GeForce RTX2080 graphics card, equipped with Intel(R) Core (TM) i7-8700 @3.20 GHz CPU processor.

In this study, the initial learning rate of all CNN classification models was set to 0.001, the Batch Size of training samples is set to 4, and the number of iterations (epoch) was set to 400. The optimization algorithm is Adam, and the loss function is the Cross Entropy Loss. During the training process, early stopping is set to prevent the model from overfitting. If the performance of the model does not improve after 30 epochs, the training will stop.

#### Classification results of wheat lodging degree based on MobileNetV2

**FIGURE 8:**
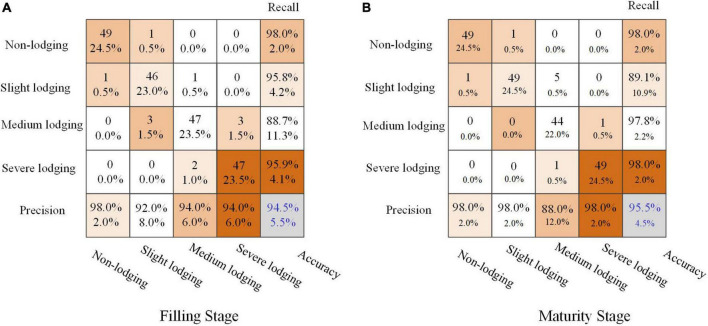
Recognition results of wheat lodging degree based on MobileNetV2. **(A)** filling stage, **(B)** maturity stage.

Figure 8 showed the recognition results of wheat lodging degrees at filling and maturity stages of wheat using the MobileNetV2 model. The point cloud dimensionality reduction data set was constructed with the dimensionality reduction images from point cloud obtained using the inverse distance weighted interpolation method, which was used to classify the lodging degree of wheat.

The accuracy for the filling and maturity stages of wheat could reach 94.5 and 95.5%. Especially in the filling stage of wheat, the classification accuracy of different lodging degrees was more than 90%. The classification results for the moderate lodging degree of wheat at maturity were slightly worse. The possible reason was that the clarity of the original data boundary of the maturity of wheat was poor, which led to wrong judgment of the data. Overall, the classification results based on dimensionality reduction images from point cloud data were better.

## Discussion

### Comparison results of different interpolation methods

To compare the effects of different interpolation methods on the lodging degree of wheat fields, the dimensionality reduction of point cloud data in wheat fields based on different interpolation methods was carried out, including local linear embedding, bitonal spline interpolation, and inverse distance weighted interpolation. From each of the four lodging degrees of wheat fields, 10 wheat plots were selected for point cloud interpolation method, and three error indexes of MAE, SD and Median were used to evaluate different interpolation methods. The average value of the error was taken as the interpolation error of different interpolation methods in different periods as the evaluation result, and the results were shown in the Table 2.

**TABLE 2:** Error indicators based on different interpolation methods.

Method	Data	MAE/mm	SD/mm	Median/mm
Local linear embedding	Filling	0.817	1.289	0.597
	Maturity	0.879	1.364	0.673
Biharmonic spline interpolation	Filling	0.572	0.863	0.331
	Maturity	0.624	0.928	0.374
Inverse distance weighted interpolation	Filling	0.412	0.754	0.216
	Maturity	0.428	0.785	0.243

As could be seen from Table 2, the error of the interpolation method of wheat field point cloud data, MAE, SD and Median were 0.412–0.817, 0.754–1.289, and0.216–0.597 for the Filling period, MAE, SD and Median were 0.428–0.879, 0.785–1.364, and 0.243–0.673 for the maturity period.

**FIGURE 9:**
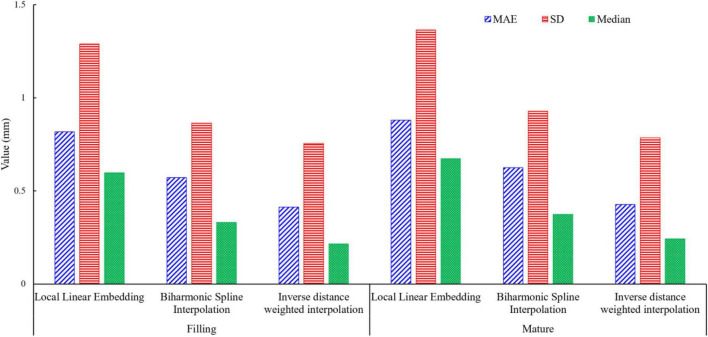
Comparison of interpolation precision.

It could be seen from Figure 9 that the three evaluation indicators of the error based on the inverse distance weighted interpolation method, such as MAE, SD, and Median, were smaller than those of other interpolation methods, no matter it was the grouting period or the maturity period. Compared with Biharmonic Spline Interpolation and Biharmonic Spline Interpolation, the error based on the Inverse distance weighted interpolation method reduced by 49.6 and 28% of MAE, 41.5, and 12.6% of SD, 63.8 and 34.7% of Median for the filling period, 53.1 and 34% of MAE, 44.7 and 18.8%, 67.9 and 42.2% of Median for maturity period. Experiments showed that the method based on inverse distance weighted interpolation had lower errors in processing wheat field lodging point cloud data in different periods.

Studies have shown that spatial interpolation methods have proven to be an important technique in point cloud data preprocessing (Liu S. F. et al., 2018). In addition to the three interpolation methods used in this study, Agüera-Vega et al. (2020) also compared inverse distance weighting (IDW), multiple quadratic radial basis functions (MRBF), kriging (KR) and linear interpolation triangulation (TLI) in processing point clouds extracted from drone images, which shows that the interpolation method has great potential, especially the point cloud data has been widely used. Therefore, more interpolation methods can be tried in future research work, aiming to provide the application efficiency of point cloud data (Agüera-Vega et al., 2020).

### Comparison of classification results using different models

To verify the performance of wheat lodging classification based on the point cloud dimensionality reduction method, data sets of different growth periods were used, including dimensionality reduction images from point cloud based on the inverse distance weighted interpolation method of wheat fields at the filling and maturity stages, which were used to train and test the classification model, including AlexNet, VGG16, MobileNetV2 models. The results were shown in Table 3. It could be seen from Table 3 that for the training set, the F1-Score of the MobileNetV2 model was 9.73% higher than that of AlexNet, and 5.02% higher than that of VGG16. The precision of the MobileNetV2 model was 9.74% higher than that of AlexNet and 5.13% higher than that of VGG16 using the point cloud data of the wheat filling period. For the test set, the F1-Score of the MobileNetV2 model was 12.43% higher than that of AlexNet and 6.9% higher than that of VGG16. The Precision of the MobileNetV2 model was 12.04% higher than that of AlexNet, and 6.81% higher than that of VGG16.

**TABLE 3:** Classification accuracy (%) of wheat field lodging degree based on different methods.

Data	Methods	F1-score	Precision	F1-score	Precision
		
		Training set		Test set	
Filling	AlexNet	88.1	88.0	83.8	84.0
	VGG16	92.7	92.5	89.1	89.0
	MobileNetV2	97.6	97.5	95.7	95.5
Maturity	AlexNet	85.8	86.0	83.1	83.0
	VGG16	91.1	91.0	87.6	87.5
	MobileNetV2	96.7	96.5	94.6	94.5

For the training set, the F1-Score of the MobileNetV2 model was 11.27% higher than that of AlexNet and 5.79% higher than that of VGG16 using the point cloud data of the wheat maturity period. The precision of the MobileNetV2 model was 10.88% higher than that of AlexNet and 5.7% higher than that of VGG16. For the test set, the F1-Score of the MobileNetV2 model was 12.16% higher than that of AlexNet and 7.4% higher than that of VGG16. The precision of the MobileNetV2 model was 12.17% higher than that of AlexNet, and 7.41% higher than that of VGG16.

By comparing the experimental results of different models, it was concluded that the classification of wheat lodging based on the dimensionality reduction images from point cloud based on the MobileNetV2 model performed well in both the filling and the Maturity stage of wheat.

The research shows that based on the role of point cloud in wheat height monitoring. At present, the method of acquiring point cloud has become more and more convenient with the development of sensors. Volpato et al. (2021) successfully extracted the height of wheat from dense point clouds generated by aerial images for monitoring of wheat growth. Dense point clouds extracted from drones carrying high-resolution RGB cameras, and Ground LiDAR were successfully used to estimate crop height (Madec et al., 2017). In particular, point cloud data can be obtained conveniently and quickly through UAV, which will play an important role in promoting crop phenotype acquisition and field management.

### Dimensionality reduction result of point cloud for wheat field

**FIGURE 10:**
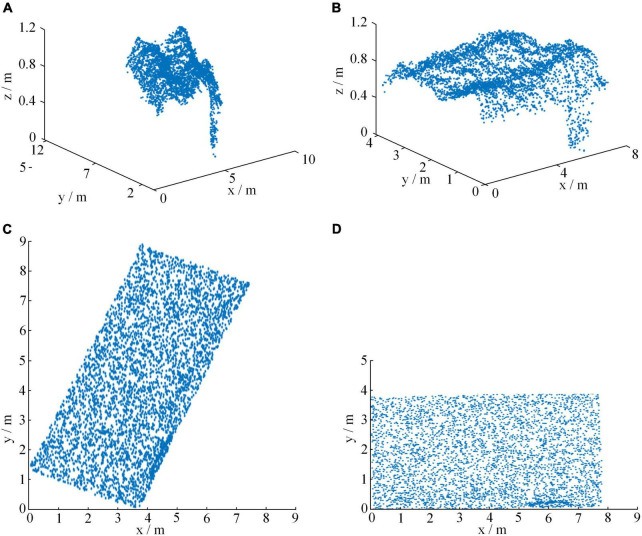
Comparison of point clouds before and after Hotelling transform. **(A)** original three-dimensional view, **(B)** original bird’s eye view, **(C)** three-dimensional view after Hotelling transform, and **(D)** bird’s eye view after Hotelling transform.

To avoid the influence of the tilt angle of the point cloud, Hotelling transform was used to coordinate the point cloud locally. Figure 10 shows the comparison results of point clouds before and after Hotelling transformation. Figures 10A,B were the three-dimensional view and the Bird’s Eye View (BEV) of the point cloud in the standard coordinate system, respectively. Figures 10C,D were the 3D view and the Bird’s Eye View (BEV) of the point cloud after Hotelling transformation, respectively. As could be seen from Figure 10, the three directions with the largest distribution of point cloud data can be found through Hotelling transformation, and then the point cloud was rotated to these three directions as a whole, so that the point cloud was regularly covered in the coordinate system.

In fact, the conversion of point clouds into 2D images has been successfully applied in many fields. For example, Li et al. (2022) transformed the point cloud into a bird’s-eye view (BEV) and verified the effect of the method on public datasets and unmanned motion platforms. Tsai et al. (2021) converted the point cloud into a bird’s-eye view, which was used as input to Faster R-CNN and YOLOv3 network architecture for tire detection. Huang et al. (2020) achieved dimensionality reduction transformation by converting 3D point cloud into 2D image through projection, which plays an important role in construction monitoring. Guo R. et al. (2021) projected point clouds onto a bird’s-eye view (BEV) for object detection. UAV-based point cloud datasets are also often used to estimate the height of plants. For example, Shin et al. (2018) estimated forest canopy height from UAV-based multispectral imagery and SfM point cloud data. There are even many studies that have successfully used point cloud datasets extracted from drone images to estimate the height of wheat, aiming to accurately monitor crop growth. For example, Song and Wang (2019) used UAV-based point cloud data to estimate the height of wheat in different periods, indicating that point cloud data has good potential for estimating crop height. Khanna et al. (2015) proposed a method for early winter wheat canopy height estimation using 3D point cloud statistical analysis. The above research shows that the application of point cloud can help farmers manage their farmland easily.

## Conclusion

In this study, a classification method of wheat lodging degree based on dimensionality reduction images of point cloud data was proposed. This method not only realized the transformation of disordered point cloud data into 2D images based on Hotelling transform and point cloud interpolation method, but also realized the classification of different lodging degrees of wheat using three CNN models, including AlexNet, VGG16, and MobileNetV2. Further, the self-built wheat point cloud data was used for testing. The results showed that the F1-score of the classification model of wheat field lodging degree based on MobileNetV2 reached 95.7% for filling period and 94.6% for maturity period, which provided the technical basis for the analysis and application of 3D point cloud data of other crops. In addition, the research results provided a scientific basis for farmland management, disaster assessment, and yield estimation. Moreover, the 3D point cloud data processing method proposed in this study will also promote the development of new technology paths for UAVs in crop remote sensing monitoring.

## Data availability statement

The original contributions presented in this study are included in the article/supplementary material, further inquiries can be directed to the corresponding author/s.

## Author contributions

YL designed the research, performed the experiments, and analyzed the data. YL, SZ, and BY wrote the manuscript. SZ and QC helped to perform the experiments. BY supervised the project and helped to design the research. All authors contributed to the article and approved the submitted version.
